# Three-level anterior cervical discectomy and fusion in elderly patients with wedge shaped tricortical autologous graft: A consecutive prospective series

**DOI:** 10.4103/0019-5413.43396

**Published:** 2008

**Authors:** Suk Ha Lee, Kwang Jun Oh, Kwang Su Yoon, Sung Tae Lee, Dilbans S Pandher

**Affiliations:** 1 Department of Orthopedics, Konkuk University Hospital, Konkuk University School of Medicine, Seoul, South Korea; 2 Department of Orthopedics, Oxford Super Specialty Hospital, Jalandhar, Punjab, India

**Keywords:** Anterior discectomy, elderly patients, plating, three-level cervical spine disease, wedge-shaped autograft

## Abstract

**Background:**

Treatment of multilevel cervical spondylotic myelopathy/radiculopathy is a matter of debate, more so in elderly patients due to compromised physiology. We evaluated the clinical and radiological results of cervical fusion, using wedge-shaped tricortical autologous iliac graft and Orion plate for three-level anterior cervical discectomy in elderly patients.

**Materials and Methods:**

Twelve elderly patients with mean age of 69.7 years (65–76 years) were treated between April 2000 and March 2005, for three-level anterior cervical discectomy and fusion, using wedge-shaped tricortical autologous iliac graft and Orion plate. Outcome was recorded clinically according to Odom's criteria and radiologically in terms of correction of lordosis angle and intervertebral disc height span at the time of bony union. The mean follow-up was 29.8 months (12–58 months).

**Results:**

All the patients had a complete recovery of clinical symptoms after surgery. Postoperative score according to Odom's criteria was excellent in six patients and good in remaining six. Bony union was achieved in all the patients with average union time of 12 weeks (8–20 weeks). The mean of sum of three segment graft height collapse was 2.50 mm (SD = 2.47). The average angle of lordosis was corrected from 18.2° (SD = 2.59°) preoperatively to 24.9° (SD = 4.54°) at the final follow-up. This improvement in the radiological findings is statistically significant (P < 0.05).

**Conclusion:**

Cervical fusion with wedge-shaped tricortical autologous iliac graft and Orion plate for three-level anterior cervical discectomy is an acceptable technique in elderly patients. It gives satisfactory results in terms of clinical outcome, predictable early solid bony union, and maintenance of disc space height along with restoration of cervical lordosis.

## INTRODUCTION

The issue of treatment choice for multilevel cervical spondylotic myelopathy continues to be debated in the literature.^1^,^2^ Controversies have developed regarding the approach (anterior/posterior), necessity of fusion, use of allograft instead of autologous bone, ideal thickness of graft,^3^ and employment of anterior cervical plating systems in addition to interbody grafting for the uncomplicated disc disease. The posterior approach is considered less complex because it does not involve the risk of dissection around neck. However, with preexisting kyphosis or instability, the multiple-level bilateral laminectomies carry potential risk of aggravating deformity and instability.^4^ Laminoplasty also cannot completely prevent the later development of kyphosis.^2^

For anterior approach, the options are anterior cervical discectomy and fusion or corpectomy and strut grafting. Corpectomy and fibular strut grafting has a reported nonunion rate of 27% for autograft and 41% for allograft.^5^ Improved fusion rates up to 97% have been reported with iliac crest strut grafting,^6^ but reconstructing defects greater than 6 cm or two vertebral bodies can be difficult and can result in permanent donor site morbidity.^7^ Anterior cervical discectomy and fusion, as originally reported by Robinson,^5^ is a highly successful procedure for the treatment of neural compression caused by disc material or osteophytes; however, the incidence of nonunion and graft collapse rises with increase in the number of segments to be fused. Age, nutritional status, and metabolic disorders in patients, as well as mechanical environment, all have been shown to affect the bony fusion. Anterior cervical plating is a useful adjunct for increasing cervical stability in the anterior cervical discectomy and fusion.^8^ It provides immediate spine stabilization after resection of bony and soft tissue elements, allowing rapid patient mobilization. It also restores normal lordotic curve and shortens fusion time because it facilitates healing by reducing micro motion^9^ and by holding bone graft under compression,^10^ and therefore significantly reducing pseudoarthrosis rates.^11^

Most of the literature on this subject is a mixed bag of single- and multilevel cases, which makes it difficult to sort out the data, and to our knowledge, there is no study reporting a series of elderly patients. The purpose of this study was to evaluate the results of anterior cervical discectomy and fusion with wedge-shaped tricortical autologous iliac graft and cervical plate in elderly patients with multilevel disc disease.

## MATERIALS AND METHODS

This consecutive prospective study consists of 12 elderly patients operated by senior authors between April 2000 and March 2005 for three-level cervical spine disease causing nerve root or cord compression at level of intervertebral disc. There were 10 men and two women with mean age of 69.7 years (range 65–76 years) [Table 1]. The mean follow-up was 29.8 months (range 12–58 months) [Table 2]. All patients were available for follow-up. Detailed clinical and radiological examination including plain X-ray and MRI was carried out in all patients preoperatively. The diagnosis was cervical spondylosis with disc herniation in 10 patients and ossified posterior longitudinal ligament (OPLL) in the remaining two. Eight patients had C3-4, 4-5, and 5-6 segment disease, while four patients had C4-5, 5-6, and 6-7 segment involvement. DEXA scan was done in all the patients to assess the bone quality. Although some of our patients were in range of osteopenia, none of our patients has osteoporosis entailing treatment.

**Table 1:** Patient demographics and final results

S. no.	Age (years)	Level	Diagnosis	Final results according to Odom's criteria
1	65	C3-4, 4-5, and 5-6	Cervical spondylopathy	Excellent
2	70	C4-5, 5-6, and 6-7	OPLL	Good
3	66	C3-4, 4-5, and 5-6	Cervical spondylopathy	Excellent
4	69	C3-4, 4-5, and 5-6	Cervical spondylopathy	Excellent
5	72	C3-4, 4-5, and 5-6	Cervical spondylopathy	Good
6	71	C4-5, 5-6, and 6-7	Cervical spondylopathy	Good
7	68	C3-4, 4-5, and 5-6	Cervical spondylopathy	Excellent
8	67	C3-4, 4-5, and 5-6	OPLL	Good
9	76	C3-4, 4-5, and 5-6	Cervical spondylopathy	Good
10	69	C4-5, 5-6, and 6-7	Cervical spondylopathy	Excellent
11	73	C3-4, 4-5, and 5-6	Cervical spondylopathy	Excellent
12	70	C4-5, 5-6, and 6-7	Cervical spondylopathy	Good
Mean (SD)	69.7 (3.08)			

**Table 2:** Radiological findings and duration of follow-up

S. no.	Cervical lordosis of three segment (°)			Sum from intervertebral span of three segments (mm)			Follow-up duration (months)
			
	Preoperative	Postoperative	Final follow-up	Preoperative	Postoperative	Final follow-up	
1	16	34	33	8	24	23	58
2	18	23	23	16	19	17	46
3	20	25	24	19	34	34	43
4	15	25	24	15	24	22	38
5	21	28	27	16	26	23	36
6	17	24	18	14	24	17	30
7	17	35	33	9	25	24	24
8	17	22	22	15	18	16	21
9	21	26	22	20	35	27	18
10	14	24	22	14	23	22	17
11	22	29	28	17	27	25	14
12	20	23	23	13	24	23	12
Mean (SD)	18.2 (2.59)	26.5 (4.25)	24.9 (4.54)	14.7 (3.52)	25.3 (5.03)	22.8 (4.90)	29.8 (14.5)

We opted for anterior approach, as there was significant loss of cervical lordosis in all cases. Left anterolateral approach to the cervical spine was performed using oblique incision along the anterior border of sternocliedomastiod muscle centered over the pivot of central involved segment. C-arm was used to locate the involved disc level. Dissection was carried out along the tissue plains, cautiously with gentle retraction to avoid postoperative tissue necrosis. Longus colli was raised laterally subperiosteally. Annulus was incised and disc material was removed using pituitary rounger laterally up to the uncinate process, which marks the safe extent of lateral decompression. The vertebral end cartilage was curetted cautiously up to the spontaneous bleeding bone, and the structural integrity of endplates was left intact. The vertebral body or ossified posterior longitudinal ligament was not eliminated. Each disc space was widened using Casper plate and the size of the graft required was measured. Autologous tricortical bone graft was harvested from anterior iliac crest and cut into wedge-shaped pieces of appropriate size to fit the gaps created. The graft was inserted between the vertebral bodies to restore the intervertebral disc space and cervical lordosis. Anterior cervical plating was done across the whole construct with Orion locking plate to provide stability till the bony fusion occurred. Postoperatively once the check radiograph of cervical spine showed that the graft and implant are placed properly, the patient is mobilized on Philadelphia Orthosis for one month.

**Figure 1:**
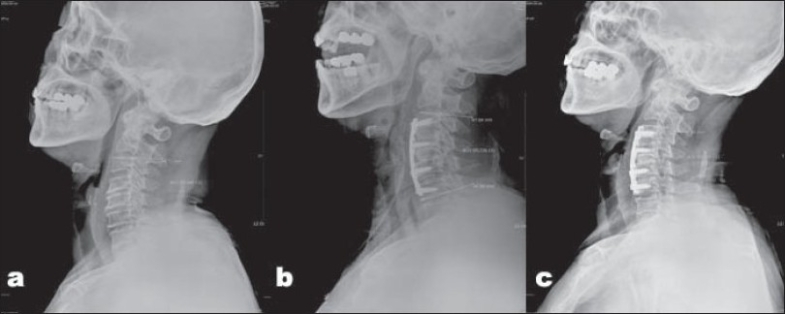
Measurement of three segment cervical lordosis. (a) Preoperative (17°) (b) Postoperative (24°) (c) At bony union (18°)

All the patients were followed up at regular monthly intervals clinically and radiologically for a minimum period of one year and thereafter at six-monthly intervals. No pain on movement, no progressive neurogenic symptoms, and no spontaneous pain in the neck at rest were considered as signs of bony fusion clinically. At each follow-up, radiographs were taken to check the proper positioning of implant, disc space height, and maintenance of correction of cervical lordosis. Fusion was judged by the absence of motion between the fused segments on flexion-extension radiograph, absence of radiolucent gap between graft and vertebral endplate, and presence of continuous bridging bony trabeculae at graft-endplate junction. The graft collapse was calculated by measuring the span between the center of the lower side of upper vertebral body and the upper side of lower vertebral body in the lateral X-ray after surgery and at bony union. The cervical lordosis was calculated by measuring the angle between the superior endplate of the upper most involved vertebra and the inferior endplate of the lower most involved vertebra [Figure 1]. Any hardware failure was recorded regardless of the significance or surgical outcome. At final follow-up, the clinical outcome was recorded according to Odom's criteria^12^ [Table 3].

**Table 3:** Showing Odom's criteria

Rating	Odom's criteria
Excellent	No complaints referable to cervical disc disease; able to continue daily occupation without impairment
Good	Intermittent discomfort related to cervical disease but not significantly cantly interfering with work
Satisfactory	Subjective improvement but physical activities limited
Poor	No improvement or worse compared with the condition before the operation

## Statistical evaluation

A Student's paired *t*-test was used to compare the preoperative and final follow-up values of three-level interbody span and local cervical lordosis. The significance was set at P < 0.05.

## RESULTS

### Clinical results

Average operative time from incision to surgical wound closure was 90 min. Excellent results were observed in six patients and good results in remaining six patients [Table 1], according to Odom's criteria. In all cases, there was noticeable reduction in neck pain and radiating pain. Myelopathy signs and symptoms disappeared in OPLL cases. The clinical and radiological union was achieved in all cases, with average union time of 12 weeks (8–20 weeks).

## Radiological results

**Figure 2:**
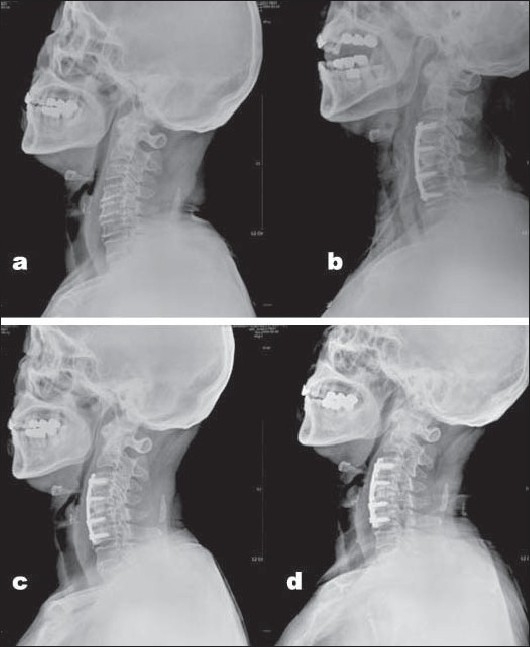
(a) Preoperative intervertebral disc heights at three involved levels (14 mm). (b) Postoperative intervertebral disc heights (24 mm). (c) At 2 months postoperatively screw loosening—halo notie around lower most screw. (d) At 3 months solid bony Union apparent (sum of three-level disc height = 17 mm)

The mean of sum of three-level interbody span was 14.7 mm (SD = 3.52) preoperatively, which increased to 25.3 (SD = 5.03 mm) postoperatively and the improvement was maintained at the final follow-up (mean = 22.8 ± 4.90 mm) [Table 2]. The improvement in interbody span at final follow-up is statistically significant according to Student's paired *t*-test (P < 0.05). The cervical lordosis was restored postoperatively to the mean value of 26.5° (SD = 4.25) from 18.2° (SD = 2.59) preoperatively. The correction in lordosis was maintained at the final follow-up with a mean value 24.9° (SD = 4.54°) [Table 2]. The correction in cervical lordosis angle at final follow-up is statistically significant according to Student's paired *t*-test (P < 0.05). The mean of the sum of three segment graft height collapse was 2.50 mm (SD = 2.47, median 2 mm). The value is apparently high because of 7 and 8 mm collapse in at 3 months after surgery in two patients, but they did not have any clinical symptoms and bony fusion was achieved at final follow-up [Figure 2].

## Complications

We had some complications like the partial loosening of screw manifested as radiolucent halo around screw threads in two cases. However, the patient had no clinical symptoms to require specific management because there was no retropulsion of screw, plate, or bone graft. Both cases progressed to solid bony union at final follow-up. Three patients complained lasting pain at the graft harvest site, which subsided over a period of six weeks. No patient in our study had neurological deficit or any other surgery-related complication as a result of surgical procedure.

## DISCUSSION

The results of multilevel anterior cervical interbody fusion are generally unsatisfactory owing to various reasons such as pseudoarthrosis, instability, graft dislodgement, and degenerative changes at the adjacent segments.^13^–^15^ The union rate is somewhat inversely proportional to the number of segments fused, with almost 100% union reported for single-segment fusion and more than 50% nonunion in multisegment fusions.^13^,^14^,^16^ This has led to the search for the effect of size, shape, type of bone, and additional treatment methods of the grafted part for Robinson's fusion technique to reduce the incidence of nonunion.^3^,^16^,^17^

Bohlman *et al*,^16^ made burr holes in the vertebral endplates before inserting the graft to increase the vascular access to graft to further augment the fusion rates. For similar reasons, Emery *et al*,^17^ used the grinder to erode the subchondral bone deep to cancellous portion of vertebra and made box-shaped gutter to secure the graft stability and further enhance union. The union rates reported were 95.6% for the single-segment fusion and 56% for three segment fusion.^14^ They recommended use of curette to remove the cartilage covering the endplate instead of grinder, taking into account the excessive bone loss as a probable cause of collapse. We curetted the endplate cartilage up to the spontaneous bleeding bone, while cautiously maintaining the structural integrity of endplates. In current series, vertebral body or ossified posterior longitudinal ligament was not eliminated unless directly pressing over neural structures, as significant percentage of osteophytes are known to be resorbed spontaneously in the presence of stable interbody fusion.^18^

Grafted bone plays a role of spacer and secures the size of neural foramen to prevent nerve compression.^15^ Although ideal thickness of the graft is reported to be directly related to preoperative baseline disc height,^3^,^4^ we believe that the intraoperative measurement of stretched disc space is the best guide for graft thickness required. Ahn *et al*,^19^ suggested that the most efficient surgical technique to restore the cervical lordosis was to insert a wedge-shaped autologous tricortical graft. Failure to restore cervical lordosis with bone graft could lead to progressive kyphotic deformation, which plays a significant role in nonunion.^20^ The iliac bone tricortical graft with adequate cortical and cancellous bone is best suited for cervical fusion.^21^ The union rate is low with allograft as compared with autograft, and its strength fails to meet the standards to maintain the disc space and neural foramen width.^18^,^22^ We used autologous wedge-shaped tricortical graft harvested from the anterior iliac crest, which we presume is an important reason for the high union rate in our series. Although minor complications like donor site pain were reported, the symptoms subsided within few weeks postoperatively.

The stretching of the ligaments initially generated by the distraction and hyperextension of the segments to insert a graft larger than the resting height of the disc space locks the graft in position with the compressive forces maintained by the stretched lateral and remaining anterior cervical segmental ligaments.^11^ But, the compressive forces are unbalanced by the soft tissue stripping done to expose multiple level discs. These altered biomechanics probably play a significant role in the graft collapse, as the contact stress at graft-body interfaces increases with an increase in number of operative levels.^23^ Under given circumstances, plating enhances the internal stabilization achieved by the graft and might help in decreasing these stresses. We used the Orion metal plate in all cases. The Orion anterior cervical plate has certain advantages over the other plating system, since screws may be placed in a convergent or divergent manner, and unicortical and bicortical screw purchase may be achieved. The amount of lordosis present in the Orion anterior cervical plate is acceptable in most instances; however, the lordosis may be altered by gently bending the plate if necessary. An anterior locking screw is threaded into the plate to prevent the bone screws from backing out.

Any bony spur on the anterior surface was cautiously removed to provide close contact between the vertebral body and plate for secure fixation. Caspar *et al*,^11^ based on retrospective analysis of 356 cases of one or two segment fusions asserted that the plating reduced the re-surgery rate. The main argument stated against plating is that any benefits to the patient as a result of plate stabilization would be lost by plate-related complications such as screw loosening, hardware breakage, or pain caused by the presence of hardware requiring revision or removal. We had two cases with screw loosening, apparent on X-ray film as radiolucent shadow around screw threads at second and third month postoperatively; however, solid bony union resulted at the final follow-up without any clinical symptoms because there was no retropulsion of screw, plate, or bone graft. Natio *et al*,^24^ reported 8% incidence of screw loosening, but there were no clinical symptoms.

Long fusions leave few mobile segments and degenerative changes in the adjacent segments are not unexpected. As the current study reports the results in elderly subjects with the mean age of 69.7 years, all the patients had some preexistent degenerative changes at the adjacent segments. We did not observe any worsening of symptoms or progression of radiological changes at adjacent segments on follow-up, because the duration of the follow-up is not sufficient to comment on any benefit of this at present. The major limitation of our study is the small number of cases; however, considering the comparatively low incidence of multilevel disease and the specific age group, we believe that the number of patients is sufficient to report our experience.

From the literature,^2^ the anterior cervical spine surgery is associated with more complications than laminoplasty or laminectomy such as increased risk of injury to vital structures of neck, wound infection, pseudoarthrosis, graft resorption, vocal cord paralysis, and difficulty in swallowing^5^,^24^ along with complications related to internal fixation like screw breakage,^25^ loosening, and dislodgement.^24^ But, the experience of the surgeon and adequate knowledge of neck anatomy can help lessen the chance of such complications. It can be considered as a stable surgical technique to form a solid bony union, and we recommend a multicenter study involving a larger number of patients.

## CONCLUSION

Anterior decompression and fusion for the multilevel cervical spondylopathy is an effective procedure. Autologous wedge-shaped tricortical iliac graft and additional fixation with Orion plate has produced good results in elderly patients with three segment disease. The most important factor for good results is the meticulous surgical technique, properly shaped adequate size graft further stabilized by pre-bent plate, avoiding extreme distraction of intervertebral disc space.
